# Case Report: Furmonertinib dose escalation in heavily pretreated EGFR-mutant lung adenocarcinoma with diffuse brain metastases

**DOI:** 10.3389/fonc.2026.1782198

**Published:** 2026-02-27

**Authors:** Zian Jin, Changhong Dong, Kaiyuan Hui, Xiaodong Jiang

**Affiliations:** Department of Oncology, The Affiliated Lianyungang Hospital of Xuzhou Medical University, Lianyungang, Jiangsu, China

**Keywords:** diffuse brain metastases, dose escalation, EGFR L858R mutation, furmonertinib, non-small cell lung cancer

## Abstract

Furmonertinib, a third-generation epidermal growth factor receptor (EGFR) tyrosine kinase inhibitor, has demonstrated systemic and central nervous system (CNS) antitumor activity in patients with EGFR-mutant non-small cell lung cancer (NSCLC); however, evidence supporting its use in patients with diffuse brain metastases after multiple lines of therapy and very poor performance status remains limited. Here, we report the case of a 53-year-old man with EGFR L858R-mutant stage IV lung adenocarcinoma who developed multifocal progression involving the lungs, liver, bones, and brain after multiple prior treatments. At admission, he had diffuse brain metastases and an Eastern Cooperative Oncology Group (ECOG) performance status of 4, and he was unable to undergo whole-brain radiotherapy because of impaired consciousness. Dose-escalated furmonertinib was initiated and led to marked relief of neurological and respiratory symptoms within a few days. Subsequent imaging assessments showed sustained clinical benefit, with improvement in performance status and activities of daily living. This case suggests that, in EGFR-mutant advanced NSCLC with extensive CNS progression after multiline treatment failure in whom radiotherapy is not feasible, high-dose furmonertinib may represent a potential salvage option and may help inform individualized treatment strategies in this high-risk population. This single case is hypothesis-generating and larger cohorts/prospective studies are needed to confirm efficacy, safety, and appropriate patient selection.

## Introduction

Lung cancer remains one of the leading causes of cancer incidence and mortality worldwide, and non-small cell lung cancer (NSCLC) accounts for approximately 80%–85% of all lung cancers (1). In Asian populations, lung adenocarcinoma harboring epidermal growth factor receptor (EGFR) mutations is relatively common (2). The introduction of third-generation EGFR tyrosine kinase inhibitors (EGFR-TKIs) has significantly improved outcomes in this subgroup, particularly in patients with brain metastases, in whom these agents have demonstrated superior CNS control compared with earlier-generation TKIs (3). Nevertheless, with increasing lines of therapy, most patients ultimately experience systemic and CNS progression. Prior studies have reported that approximately 20%–40% of patients with NSCLC develop brain metastases during the course of disease, and the risk is substantially higher in patients with EGFR mutations than in those with EGFR wild-type tumors (4). For patients with diffuse brain metastases and poor performance status, subsequent therapeutic options are extremely limited, and intracranial disease often drives prognosis and quality of life.

Current treatment strategies for EGFR-mutant NSCLC with brain metastases commonly include whole-brain radiotherapy or stereotactic radiotherapy in combination with EGFR-TKIs. Compared with first- and second-generation agents, third-generation EGFR-TKIs penetrate the blood–brain barrier more effectively and have shown advantages in intracranial disease control and delaying CNS progression. Studies of osimertinib, in particular, have demonstrated a high intracranial objective response rate and meaningful benefits in CNS progression-free survival (PFS) (5). However, radiotherapy is often not feasible in patients with extremely poor performance status (e.g., ECOG performance status ≥3), impaired consciousness, or an inability to tolerate radiotherapy in the supine position. In such cases, selecting a systemic therapy with manageable toxicity, convenient administration, and established CNS activity remains a practical clinical challenge (6).

Furmonertinib is an oral, irreversible third-generation EGFR-TKI with high selectivity, a relatively wide therapeutic window, and favorable CNS penetration. Previous studies have shown systemic and intracranial antitumor activity in advanced NSCLC harboring common sensitizing EGFR mutations as well as certain uncommon mutations (7), with overall manageable toxicity across a daily dose range of 80–240 mg (6). In addition, retrospective reports have suggested that re-treatment with furmonertinib at 160 mg after failure of prior third-generation EGFR-TKI therapy may still achieve renewed responses in both systemic and intracranial lesions in some patients, supporting a potential role for a high-dose escalation strategy (8). However, evidence remains scarce in patients with multiline treatment failure, diffuse brain metastases, profoundly impaired performance status, and ineligibility for radiotherapy. Against this background, we report a case of advanced lung adenocarcinoma after multiple lines of therapy treated with dose-escalated furmonertinib.

## Case presentation

The patient was a 53-year-old man. In September 2023, he presented to a local hospital with chest tightness and cough. Chest and abdominal computed tomography (CT) revealed multiple nodular lesions in both lungs accompanied by bilateral hilar and mediastinal lymphadenopathy, suggestive of metastatic disease. Positron emission tomography/CT (PET/CT) demonstrated enlarged lymph nodes in the bilateral supraclavicular regions, mediastinum, bilateral hila, hepatogastric ligament, and retroperitoneum. Multiple pulmonary nodules were observed in both lungs, along with multiple abnormal skeletal lesions. A subcapsular nodular lesion was also noted at the dome of the right hepatic lobe. Overall, these findings were considered consistent with a primary lung malignancy with widespread metastases. In October 2023, a percutaneous biopsy of a right lung lesion was performed, and histopathology confirmed lung adenocarcinoma. Based on imaging, the disease was staged as cTxN3M1 (stage IV). Molecular testing identified an EGFR L858R point mutation, and T790M was negative.

First-line targeted therapy with osimertinib (80 mg once daily) was initiated in October 2023. Follow-up chest CT scans in January and April 2024 showed a marked reduction in bilateral pulmonary lesions, and the response was assessed as partial response (PR). His symptoms, including cough and chest tightness, improved, and he was largely able to perform activities of daily living independently.

In August 2024, the patient re-presented with cough and dyspnea. Chest CT revealed interstitial inflammatory changes and multiple pulmonary nodules in both lungs, with an increased disease burden compared with prior imaging, suggestive of tumor progression with concomitant infection. Osimertinib was discontinued, and EGFR-TKI therapy was continued with aumolertinib (110 mg once daily).

In December 2024, chest CT demonstrated a mass lesion in the left upper lobe with obstructive inflammation, multiple nodules in both lungs, and multiple hyperdense osseous lesions involving the sternum and the cervical, thoracic, and lumbar spine, indicating further progression of pulmonary disease and bone metastases.

On January 4 and January 25, 2025, the patient received two cycles of chemotherapy consisting of albumin-bound paclitaxel (0.4 g on day 1) plus cisplatin (60 mg on day 1) in combination with bevacizumab (0.4 g on day 1). Denosumab was also administered for the management of bone involvement. On January 27, he was admitted for massive hemoptysis, which improved after percutaneous bronchial artery embolization.

In February 2025, a follow-up brain MRI performed at an outside hospital revealed small intracranial nodular lesions suggestive of brain metastases; given the small lesion size, radiotherapy was deferred. Subsequently, he underwent two cycles of interventional chemotherapy with docetaxel (100 mg on day 1) on February 21 and March 17. During treatment, he gradually developed neurological symptoms, including gait instability, dizziness, headache, memory decline, and slurred speech.

Approximately 10 days before admission, his neurological symptoms progressively worsened and were accompanied by lethargy, slowed responses, and reduced bladder and bowel control. On April 11, 2025, he was admitted to our hospital for progressive neurological deterioration with impaired consciousness. On admission, his general condition was extremely poor; he could respond only briefly to simple questions, with a decreased level of consciousness and an ECOG performance status of 4. Arterial blood gas analysis indicated mild hypoxemia and hyponatremia, accompanied by mild anemia.

**Figure 1:**
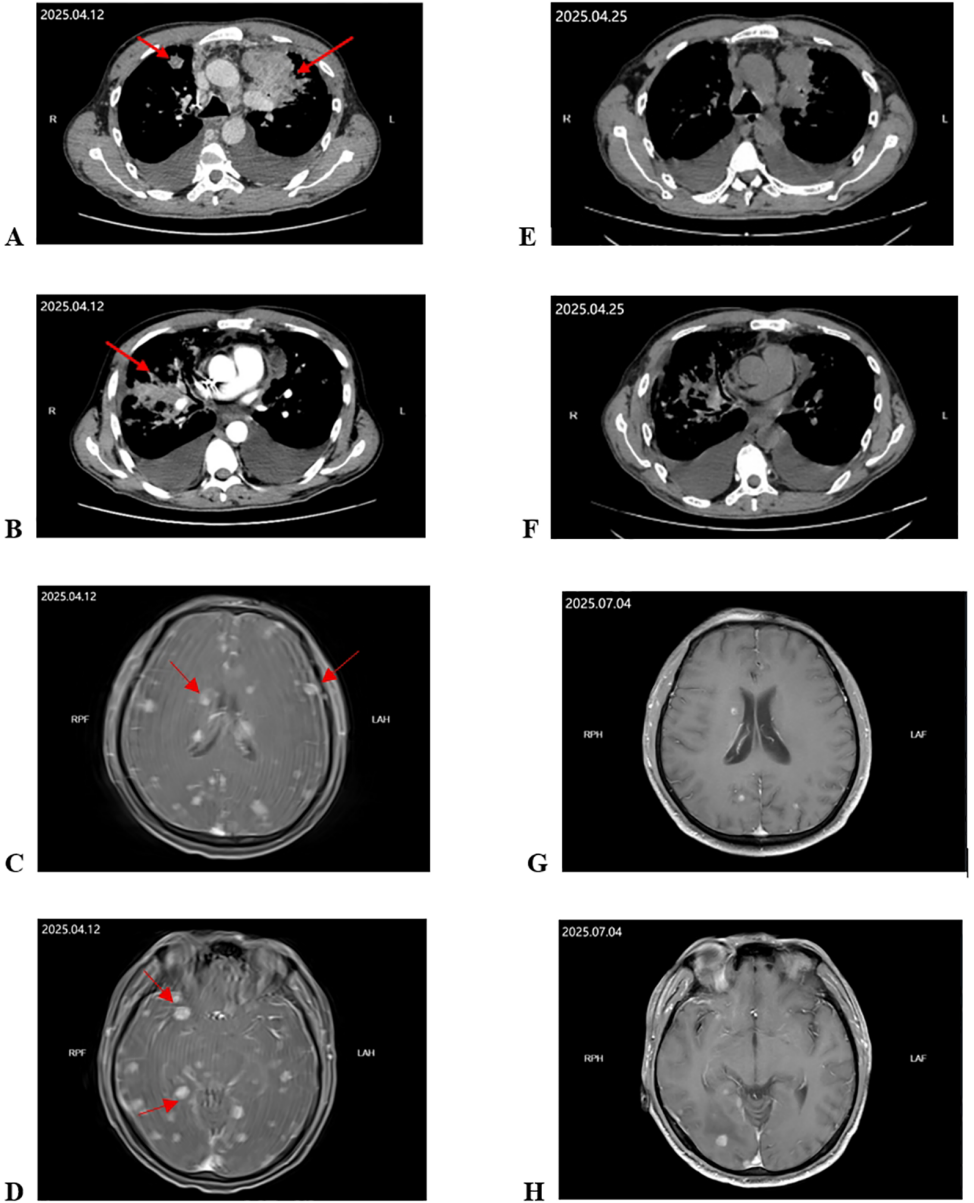
Changes in target lesion during treatment. **(A–D)** CT scan and MRI before treatment. **(E, F)** CT scan obtained 13 days after treatment. **(G, H)** MRI obtained nearly 3 months after treatment. The red arrows mark the target lesions.

On April 12, contrast-enhanced chest CT demonstrated lung cancer with multiple bilateral pulmonary metastases, marked progression of carcinomatous lymphangitis, increased bilateral pleural effusions, and multiple enlarged lymph nodes in the mediastinum and cardiophrenic angles (**Figures 1A, B**). Multiple intrahepatic metastases, left adrenal metastasis, and widespread bone metastases were also identified. On April 13, contrast-enhanced brain MRI (image quality was limited due to the patient’s poor condition) revealed multiple nodular enhancing lesions in both cerebral hemispheres and the cerebellum, consistent with multiple brain metastases (**Figures 1C, D**).

After admission, mannitol and corticosteroids were administered for suspected cerebral edema (standard supportive regimen). He also received supportive care, including continuous electrocardiographic monitoring and supplemental oxygen. Meanwhile, ultrasound-guided left-sided thoracentesis with catheter drainage was performed. On April 21, intrapleural chemotherapy with cisplatin (60 mg) was administered via the pleural drainage catheter.

Based on the brain MRI findings indicating diffuse brain metastases, whole-brain radiotherapy (WBRT) was considered; however, the patient had impaired consciousness and profound functional dependence (ECOG 4), could not cooperate with simulation, and could not safely maintain a supine position for treatment; therefore, radiotherapy was not feasible. To achieve systemic control of both intracranial and extracranial disease, the multidisciplinary team considered further oral targeted therapy.

Given the presence of an EGFR L858R mutation and disease progression after multiple prior lines of treatment—including osimertinib, aumolertinib, platinum-based chemotherapy, and interventional chemotherapy—and the coexistence of diffuse brain metastases with widespread systemic metastases, the critical illness and extremely poor prognosis were discussed in detail with the patient’s family. Subsequently, oral furmonertinib at a dose of 160 mg once daily was initiated on April 19, 2025, as a salvage systemic treatment.

Within several days of dose-escalated furmonertinib, the patient experienced marked improvement in dizziness, headache, and dyspnea. His mental status and oral intake improved, and bladder and bowel control gradually recovered. A follow-up chest CT on April 25, compared with that on April 12, showed varying degrees of shrinkage of the left upper lobe mass and multiple bilateral pulmonary metastatic lesions, with decreased bilateral pleural effusions, indicating a favorable pulmonary response (**Figures 1E, F**). After clinical stabilization, he was discharged and continued furmonertinib with outpatient follow-up.

On July 4, 2025, contrast-enhanced brain MRI demonstrated that multiple pre-existing nodular or short linear enhancing lesions in the bilateral frontal, parietal, temporal, and occipital lobes, periventricular regions, basal ganglia, brainstem, and cerebellar hemispheres had partially decreased in size or disappeared compared with the MRI performed on April 13 (e.g., a right occipital lesion measuring up to approximately 8 × 8 mm), suggesting substantial intracranial disease control (**Figures 1G, H**). At follow-up, his neurological function and activities of daily living were markedly improved compared with those at admission. Meanwhile, repeat systemic imaging showed stable hepatic and osseous metastatic lesions. However, during subsequent follow-up, the patient developed progressive intrathoracic disease with massive pleural effusion, accompanied by a marked decline in performance status. Despite supportive care, he ultimately died of multi-organ failure. A comprehensive summary of the patient’s treatment timeline and clinical course is provided in **Table 1**.

**Table 1:** Treatment timeline and clinical course.

Date	Treatment/intervention	Radiologic assessment	Clinical symptoms	ECOG performance status
Oct 2023	Initiated osimertinib 80 mg once daily	Partial response (PR) of pulmonary lesions	Improvement in cough and chest tightness	1
Aug 2024	Switched to aumolertinib 110 mg once daily	Pulmonary progression with concomitant lung infection	Worsening cough and dyspnea	2
Jan 2025	Chemotherapy (albumin-bound paclitaxel + cisplatin + bevacizumab)	Radiologic progression of bone metastases	Massive hemoptysis	3
Apr 12, 2025	Hospital admission; whole-brain radiotherapy not feasible	Diffuse brain metastases on MRI	Impaired consciousness and urinary/fecal incontinence	4
Apr 19, 2025	Initiated furmonertinib 160 mg once daily	Reduction in pulmonary lesions on imaging	Rapid improvement in neurological symptoms	3 → 2
Jul 4, 2025	Continued furmonertinib therapy	Intracranial lesions reduced, some resolved on MRI	Recovery of activities of daily living	2

## Discussion

This patient had advanced lung adenocarcinoma harboring an EGFR L858R mutation and achieved a partial response to first-line osimertinib. After multiple subsequent lines of therapy—including aumolertinib, platinum-based chemotherapy combined with bevacizumab, and interventional chemotherapy—he developed multifocal progression involving the lungs, bones, liver, and brain and was close to an end-stage condition at admission. In routine guideline-based practice, best supportive care is often prioritized for patients with advanced NSCLC and profoundly impaired performance status, and whether to continue systemic antitumor therapy in this setting remains highly controversial.

In this case, whole-brain radiotherapy was not feasible because of impaired consciousness and an inability to tolerate prolonged supine positioning. Therefore, dose-escalated oral furmonertinib (160 mg once daily) was attempted. Neurological and respiratory symptoms improved markedly within a short period, accompanied by synchronous shrinkage of both pulmonary and intracranial lesions. These findings suggest that high-dose furmonertinib may have potential salvage value in EGFR-mutant lung adenocarcinoma with multiline resistance and concomitant CNS progression. Notably, despite the rapid intracranial and symptomatic improvement, the patient subsequently developed predominant intrathoracic progression with massive pleural effusion and a marked decline in performance status, and he ultimately died of multi-organ failure. This clinical course underscores that short-term CNS responses do not necessarily translate into durable systemic disease control in heavily pretreated advanced NSCLC.

For patients with EGFR-mutant advanced NSCLC, EGFR-TKIs substantially prolong PFS; however, acquired resistance is almost inevitable (9). Subsequent treatment options remain limited, particularly in patients with multiline treatment failure and poor performance status, in whom chemotherapy combined with anti-angiogenic agents or immunotherapy is often associated with considerable toxicity and offers limited intracranial disease control. Furmonertinib is an oral, irreversible third-generation EGFR-TKI with high selectivity, a relatively wide therapeutic window, and favorable penetration across the blood–brain barrier. The phase III FURLONG study demonstrated that, in treatment-naïve patients with advanced NSCLC harboring sensitizing EGFR mutations, first-line furmonertinib significantly prolonged PFS compared with gefitinib (10) and improved CNS-PFS and intracranial response rates in patients with baseline CNS metastases (11). A phase II study in patients with EGFR T790M-positive disease also confirmed that furmonertinib at the standard dose achieved a high intracranial response rate and a prolonged CNS-PFS (12). Furthermore, retrospective and real-world studies have suggested that, in high-risk populations such as those with brain metastases or leptomeningeal metastases, dose escalation of furmonertinib to 160–240 mg/day—while maintaining overall manageable toxicity—may further increase intracranial response rates and prolong CNS-PFS, suggesting a potential dose–effect relationship (13). Collectively, these data provide a clinical rationale for adopting furmonertinib 160 mg as a salvage strategy after multiline treatment failure in the present case, rather than a purely empirical attempt.

Key features of this case include the high disease burden, extensive metastatic involvement, and ECOG performance status of 4 after multiple systemic therapies, including two third-generation EGFR-TKIs and platinum-based chemotherapy. Such patients are often considered end-stage and deemed suitable only for palliative and supportive care. After careful assessment of the risk–benefit balance and thorough discussion with the patient’s family, high-dose furmonertinib was selected because of its relatively manageable toxicity profile and established CNS activity. This approach led to rapid symptomatic relief and substantial radiographic tumor regression. This case is hypothesis-generating and suggests that dose-escalated furmonertinib may be considered as a salvage option in carefully selected patients with EGFR-mutant NSCLC, extensive CNS involvement, and ineligibility for radiotherapy. Prospective studies and larger real-world cohorts are required to confirm efficacy, safety, and appropriate patient selection. From a pharmacological perspective, furmonertinib and its active metabolite are thought to exert inhibitory activity against sensitizing EGFR mutations and to achieve relatively high drug concentrations in brain tissue, providing a theoretical advantage in controlling both parenchymal and leptomeningeal metastases. In this case, systematic re-biopsy or repeat molecular profiling for resistance was not performed; therefore, the presence of specific secondary EGFR alterations or bypass pathway activation could not be determined. Nonetheless, the favorable response to dose-escalated furmonertinib after failure of multiple TKIs and chemotherapy suggests that EGFR-TKI-sensitive tumor clones may have persisted *in vivo*. Increasing the furmonertinib dose to enhance drug exposure may help suppress certain resistant subclones, thereby contributing to concurrent systemic and intracranial responses. This hypothesis, however, requires confirmation by future molecular investigations.

Several limitations should be acknowledged. First, as a single case report, the findings lack a comparator and large-sample evidence, limiting generalizability. Second, dynamic ctDNA monitoring or repeat tissue sampling was not performed, and the mechanisms of resistance remain unclear; consequently, a direct link between the observed benefit from furmonertinib dose escalation and specific molecular events cannot be established. In addition, the patient received concomitant palliative and symptomatic interventions—including pleural catheter drainage, intrapleural chemotherapy, dehydration therapy, and corticosteroids for cerebral edema—which may also have contributed, at least in part, to clinical improvement, making it difficult to completely disentangle their effects. Furthermore, although the patient’s eventual death following intrathoracic progression was documented, standardized serial radiographic assessments and molecular reassessment at progression were not performed. Therefore, the durability of systemic disease control and the biological basis of subsequent resistance remain uncertain.

In summary, this case is hypothesis-generating and suggests that, for patients with EGFR L858R-mutant advanced lung adenocarcinoma who experience multiline systemic treatment failure and present with extensive brain metastases and profoundly impaired performance status, salvage therapy with high-dose furmonertinib—after careful risk–benefit assessment and alignment with patient and family preferences—may provide short-term symptomatic and radiographic improvement when radiotherapy is not feasible. Future prospective studies and larger real-world datasets are warranted to better define the optimal candidates, dosing strategies, and safety profiles for high-dose furmonertinib after EGFR-TKI resistance, particularly in patients with CNS progression. Further research is also needed to identify predictive biomarkers to support more evidence-based, individualized treatment in this high-risk population.

## Data Availability

The datasets presented in this article are not readily available due to patient privacy and institutional restrictions. Requests to access the datasets should be directed to Zian Jin, jinzian2000@163.com.
